# Deciphering oligomeric proanthocyanidins’ dual osteoprotective mechanisms at single-cell resolution: *NR4A1*-mediated *PTGS2* suppression and β-catenin-*Runx2* activation

**DOI:** 10.3389/fimmu.2025.1679987

**Published:** 2025-11-04

**Authors:** Li Huang, Yuwei Sun, Yi Zheng, Shicheng Qiu, Jianping Zheng, Chunhan Sun, Mingwei Chen, Shaowei Zheng, Yirong Zeng

**Affiliations:** ^1^ The First Clinical Medical College of Guangzhou University of Chinese Medicine, Guangzhou, China; ^2^ Department of Orthopedics, Jiangmen Hospital of Traditional Chinese Medicine Affiliated to Jinan University, Jiangmen, China; ^3^ College of First Clinical Medicine, Shandong University of Traditional Chinese Medicine, Jinan, China; ^4^ Department of Orthopaedics, Huizhou First Hospital, Guangdong Medical University, Huizhou, China; ^5^ Department of Endocrinology, the First Affiliated Hospital of Anhui Medical University, Hefei, Anhui, China; ^6^ Department of Joint Surgery, The First Affiliated Hospital of Guangzhou University of Chinese Medicine, Guangzhou, Guangdong, China

**Keywords:** osteoporosis, oligomeric proanthocyanidin, *PTGS2*, *NR4A1*, scRNA-seq

## Abstract

**Background:**

Osteoporosis (OP), as a systemic bone disorder, has a complex pathogenesis and faces significant challenges in clinical treatment. Oligomeric proanthocyanidin (OPC), a type of natural polyphenolic flavonoid compound, demonstrates outstanding therapeutic potential due to its excellent antioxidant and anti-inflammatory properties and good safety. The breakthrough advances in single-cell RNA sequencing (scRNA-seq) technology have provided a powerful research tool for elucidating the multitarget mechanisms of OPC in the treatment of OP.

**Methods:**

This study first screened the active components of OPC leveraging the TCMSP database. The protein–protein interaction network of OPC target proteins was generated through the STRING database, and visual analysis was accomplished using the Cytoscape software. The ClusterProfiler R package and ClueGO plugin were employed for functional enrichment analysis and network visualization. At the same time, scRNA-seq data from the GEO database were integrated, and cell-type identification was attained using the Seurat tool. The differentiation trajectories of subtypes were inferred using Monocle and Slingshot software. The cell communication network was analyzed using CellChat.

**Results:**

This study utilized scRNA-seq to identify C2 *NR4A1*+ MSCs with distinct metabolic features and differentiation potential in the bone microenvironment during the early stage of OP, namely, osteopenia. The natural component OPC can precisely target this subtype and exert therapeutic effects through two mechanisms: inhibiting the transcriptional activity of *NR4A1* to suppress the expression of *PTGS2* in MSCs and simultaneously activating the β-catenin-dependent *NR4A1*–*Runx2* signaling axis to promote osteogenesis and inhibit osteoclastogenesis. These findings establish a new therapeutic paradigm of “targeting cell subtypes–multipathway regulation,” providing an important basis for the development of novel anti-OP drugs.

**Conclusion:**

Our research integrated multilevel approaches, including single-cell transcriptomics, network pharmacology, cellular experiments, and animal models, to systematically reveal the dual mechanism of OPC in treating OP. This discovery not only established C2 *NR4A1*+ MSCs as key mediators in the pathological process of OP but also clarified the molecular mechanism of multitarget synergy of natural active compounds in restoring bone homeostasis, providing a theoretical basis and practical guidance for the development of new OP therapies.

## Introduction

Osteoporosis (OP) is a systemic bone disease typified by decreased bone mass, deteriorated bone microstructure, and heightened bone fragility (1–3). It affects approximately 200 million people worldwide and causes 9 million fractures each year (4). It is the fourth most common chronic disease after cardiovascular disease, dementia, and lung cancer (5), imposing a heavy socioeconomic burden. OP is caused by factors such as aging, osteoarthritis, and estrogen insufficiency, and it mainly occurs in postmenopausal women and the elderly (6, 7). Among women over 50, the risk of fracture is as high as one in three (8).

Current treatments for OP mainly follow two major principles: anti-bone resorption and pro-bone formation. Drugs like bisphosphonates and denosumab are used to inhibit osteoclast activity, while parathyroid hormone_1-34_ (PTH_1-34_) and romosozumab are used to activate osteogenic pathways (4, 9, 10). However, existing therapies still face severe challenges in clinical application. In terms of drug safety, bisphosphonates have a 40% treatment failure rate, and long-term use may lead to atypical fractures (4, 11, 12), while romosozumab has a risk of cardiovascular events (9). Secondly, the efficacy is limited. Existing drugs cannot fully restore bone microstructure (13), and anti-resorption agents can only delay bone loss but not promote new bone formation (9). More importantly, targeted therapy faces multiple bottlenecks. The complex bone immune microenvironment makes it difficult for a single target (such as RANKL) to achieve comprehensive regulation (6). At the same time, the high heterogeneity of bone marrow mesenchymal stem cells (BMSCs) and the interference of multiple factors make it difficult to target osteogenic differentiation pathways (such as Wnt) (14). In addition, clinical needs have not been fully met. On the one hand, early diagnosis still relies on bone density testing and lacks sensitive biomarkers (15). On the other hand, individualized treatment has not been fully realized, and the application of genetic and immune typing data is insufficient (9).

Oligomeric proanthocyanidin (OPC), a type of naturally occurring polyphenolic flavonoid compound, is widely found in various plants such as grape seeds, pine bark, and hawthorn (16, 17). It is one of the most abundant polyphenols in the plant kingdom (18). Research has shown that OPC possesses significant biological activities and is renowned for its powerful antioxidant, anti-inflammatory, anticancer, and anti-aging properties (19, 20). It has been developed as a nutritional supplement and applied in various health fields (18). Notably, OPC demonstrates great therapeutic potential in various chronic diseases such as inflammation, metabolic disorders, and cardiovascular diseases (21, 22). Meanwhile, oral OPC helps regulate intestinal homeostasis (23), and its metabolites have higher concentrations and longer durations of action in the body (24). However, the mechanism of action of OPC in OP has yet to be fully elucidated, but existing studies suggest that it has a strong bone-protective effect and can successfully prevent bone mass loss brought on by ovariectomy (OVX) (25).

In recent years, single-cell RNA sequencing (scRNA-seq) technology has demonstrated significant technical advantages in the field of OP research by providing high-resolution analysis of cellular heterogeneity in the bone tissue microenvironment (4, 9). This technology can precisely identify various cell populations in the bone marrow microenvironment, particularly the specific molecular characteristics of functional subtypes such as BMSCs and osteoblasts, providing potential targets for exploring new diagnostic and therapeutic strategies. At the same time, it reveals the dynamic changes of these cell populations during the pathogenesis of OP, highlighting the complexity of the bone immune environment. In the bone microenvironment, the differentiation and activation of osteoclast precursors depend on the regulation of RANKL and various cytokines secreted by osteoblasts and immune cells (such as T cells, B cells, and macrophages) (26) and are also supported by the nutrition and inflammatory regulation provided by the vascular network constructed by endothelial cells (27). In this complex network, MSCs not only are an important source of osteoblasts but also play a core role in bone homeostasis and repair by secreting growth factors and regulating immune responses (7).

This study successfully identified a subtype of C2 *NR4A1*+ MSCs with distinct metabolic features and differentiation potential in the bone microenvironment of osteopenia using scRNA-seq. This subtype forms a specific intercellular communication network with osteoblasts through the FGF7–FGFR1 ligand receptors. More importantly, we found that the natural active component OPC can precisely target this key cell subtype and exert therapeutic effects through a dual synergistic mechanism: on the one hand, *in vitro* experiments confirmed that OPC effectively inhibits *PTGS2* expression by interfering with *NR4A1*-mediated transcriptional regulation; on the other hand, OPC activates the β-catenin-dependent *NR4A1*–*Runx2* signaling axis, promoting osteogenic differentiation while inhibiting osteoclastic activity. Animal experiments further confirmed that OPC treatment can significantly improve bone microstructure parameters and restore the balance of bone metabolism markers. These systematic discoveries not only reveal new mechanisms of OP at single-cell resolution but also, more importantly, establish a therapeutic paradigm of “precisely targeting key cell subtypes and multipathway coordinated regulation,” providing an important theoretical basis and transformation direction for the development of a new generation of OP treatment regimens based on natural products.

## Materials and methods

### Obtaining the target gene dataset of OPC

We searched for the target proteins related to the OPC drug molecule in the Traditional Chinese Medicine Systems Pharmacology (TCMSP) database. Using the UniProt (https://www.uniprot.org/) database, we performed gene conversion and standardization of the target proteins and constructed the OPC target gene dataset.

### Network construction and analysis

We constructed the protein–protein interaction (PPI) network (28) of OPC target genes using the STRING database (https://string-db.org/) and then visualized and analyzed the network using the Cytoscape software (v3.10.3) (29, 30). To learn more about the biological functions of the key targets, we carried out Gene Ontology (GO) (31, 32) and Kyoto Encyclopedia of Genes and Genomes (KEGG) (33) enrichment analyses using the ClueGO plugin (34).

### Acquisition and processing of data

The scRNA-seq data of osteopenia were sourced from the Gene Expression Omnibus (GEO) database (https://www.ncbi.nlm.nih.gov/geo/) (accession number: GSE147390). Given that the data used in our study came from a public source, it was considered unnecessary to conduct an ethical review. Data preprocessing and quality control were accomplished using R software (v4.3.3) in conjunction with the Seurat package (v4.3.0). Subsequently, high-quality cells were selected upon these strict criteria: nFeature (300-7,500), nCount (500–100,000), mitochondrial gene expression (<25% of total counts), and erythrocyte gene expression (<5% of total counts). The data were normalized via the “NormalizeData” function, and the top 2,000 highly variable genes were extracted with the “FindVariableFeatures” function (35). After data standardization using the “ScaleData” function, principal component analysis (PCA) was performed (36, 37). Finally, the top 30 principal components were selected for subsequent analysis, and data dimensionality reduction and visualization were achieved through the uniform manifold approximation and projection (UMAP) (38, 39). All analyses were completed based on single-cell data that had undergone strict quality control, ensuring the reliability of the research results.

### Cell-type identification and annotation

Cell clustering analysis was conducted via the “FindClusters” and “FindNeighbors” functions in Seurat, and the differentially expressed genes (DEGs) of each cell cluster were identified using the “FindAllMarkers” function. Subsequently, cell-type annotation was carried out by combining the CellMarker database (http://xteam.xbio.top/CellMarker/) and published literature.

### Enrichment analysis

By adopting a systematic functional enrichment analysis method, the biological significance of DEGs was comprehensively analyzed. Firstly, GO (40, 41) analysis was performed with the ClusterProfiler R package (v4.6.2) (42–44), and the DEGs were functionally classified at the biological process (BP) level (45). At the same time, the KEGG database was combined to conduct metabolic pathway enrichment analysis, thereby systematically clarifying the functional characteristics of the DEGs at the biological process and metabolic pathway levels. On this basis, gene set enrichment analysis (GSEA) (46, 47) was further employed to conduct weighted analysis on predefined gene sets. By computing the enrichment score of the gene sets in the expression profile, gene sets showing coordinated expression changes in specific biological processes were identified.

### Analysis of pseudotime and lineage trajectory of MSC subtypes

To better comprehend the differentiation dynamics and developmental trajectory of MSCs, we employed Monocle (v2.24.0) (48, 49) to construct a pseudotime trajectory and identify distinct states revealed by it. Through the chronological sorting, we revealed the regularity of the continuous evolution of cell states. To further analyze the lineage relationships among different subtypes, we utilized the “getLineages” function of Slingshot (v2.6.0) (37, 50) to establish a lineage architecture based on the minimum spanning tree (MST) and then fitted smooth differentiation trajectory curves using the “getCurves” function. The biological background of MSC subtypes and the expression of their respective marker genes were incorporated in defining the starting and ending points of the trajectories.

### Analysis of the intercellular communication network

We used the CellChat package (v1.6.1) (51, 52) to conduct quantitative analysis of the intercellular interaction network based on scRNA-seq data. Based on the principle of ligand–receptor interaction, relevant signaling pathways and receptor–ligand pairs were identified through the CellChatDB database (http://www.cellchat.org/), applying a *P*-value threshold of 0.05.

### Transcriptional regulation analysis

In order to systematically analyze the gene regulatory network characteristics of MSC subtypes, we used the pySCENIC package (v0.10.0) (53–55) in Python (v3.7) for single-cell regulatory network analysis. With GRNBoost, co-expression modules involving transcription factors (TFs) and their predicted target genes can be inferred. Additionally, we constructed an AUCell matrix for this study to reveal the regulatory mechanisms of key TFs.

### Animal model preparation and experimental design

Osteoblast-specific β-catenin conditional knockout mice (*Ctnnb1^flox/flox^*; *Col1a1-Cre*) were established via Cre-loxP recombination. Floxed *Ctnnb1* mice (Stock No. 004152) and *Col1a1-Cre* transgenic mice (Stock No. 016237), both obtained from the Cancer Hospital, Chinese Academy of Medical Sciences, were intercrossed. Heterozygous offspring (*Ctnnb1^flox/+^*; *Col1a1-Cre*) were bred to generate experimental mice (*Ctnnb1^flox/flox^*; *Col1a1-Cre*) and Cre-negative littermates as controls. Genotyping was performed on genomic DNA isolated from tail biopsies using PCR with specific primers for the floxed *Ctnnb1* allele and *Col1a1-Cre* transgene. All animal procedures were reviewed and approved by the Institutional Animal Care and Use Committee of Anhui Medical University and conducted under SPF conditions in compliance with ARRIVE guidelines.

Eight-week-old female C57BL/6 mice were purchased from the Animal Laboratory Center, Anhui Medical University and acclimated under specific pathogen-free conditions. Age-matched β-catenin conditional knockout mice (osteoblast-specific, Cre-loxP system) were used as genetic controls and subjected to the same experimental procedures. Mice were randomly assigned to the following groups: sham, OVX, OVX + vehicle, OVX + OPC (20 mg/kg), and OVX + OPC (50 mg/kg). Bilateral OVX was performed under isoflurane anesthesia during the second week, while sham-operated mice underwent identical procedures without ovary removal. Postoperative recovery lasted 2 weeks, during which OVX model establishment was confirmed. From the fifth week onward, mice in treatment groups received daily oral gavage of OPC at either 20 or 50 mg/kg; OVX control mice were given an equivalent volume of vehicle. Sham mice received no treatment. Drug administration continued for 12 weeks. At week 17, all mice were humanely euthanized by CO_2_ inhalation (20% chamber volume per minute flow rate, gradually increased until loss of consciousness), followed by cervical dislocation to ensure death, in accordance with the AVMA Guidelines for the Euthanasia of Animals (2020).

### Mesenchymal stem cell isolation and culture

Human mesenchymal stem cells (hMSCs) were purchased from Cyagen Biosciences (Santa Clara, CA, USA) and cultured in low-glucose DMEM (Thermo Fisher Scientific, Waltham, MA, USA, Cat. No. 11885-084) supplemented with 10% fetal bovine serum (Gibco, Waltham, MA, USA, Cat. No. 10099-141) and 1% penicillin–streptomycin (Gibco, Waltham, MA, USA, Cat. No. 15140-122) at 37°C in a humidified incubator with 5% CO_2_. For murine primary MSC isolation, C57BL/6 mice were euthanized, and femurs and tibias were harvested under sterile conditions. Bone marrow was flushed with PBS (Thermo Fisher Scientific, Waltham, MA, USA, Cat. No. 10010-023) using a 26G needle, and the cell suspension was passed through a 70-µm cell strainer (Corning Incorporated, Corning, New York, USA, Cat. No. 352350). Cells were plated in culture flasks with complete MSC medium and incubated at 37°C with 5% CO_2_. Non-adherent cells were removed after 48 h, and the medium was replaced every 2–3 days. When cells reached 70%–80% confluence, they were passaged using 0.25% trypsin–EDTA (Thermo Fisher Scientific, Waltham, MA, USA, Cat. No. 25200-056) for expansion and downstream applications.

### CCK-8 assay

hMSCs were cultured in low-glucose DMEM (Thermo Fisher Scientific, Waltham, MA, USA, Cat. No. 11885-084) supplemented with 10% fetal bovine serum (Gibco, Waltham, MA, USA, Cat. No. 10099-141) and 1% penicillin–streptomycin (Gibco, Waltham, MA, USA, Cat. No. 15140-122) at 37°C in a humidified incubator with 5% CO_2_. Cell viability was assessed using the Cell Counting Kit-8 (CCK-8, Abcam, Cambridge, United Kingdom, Cat. No. ab228554), adhering to the manufacturer’s protocol. MSCs were seeded in 96-well plates at a density of 5 × 10³ cells per well in 100 μL of complete medium. After treatment with the indicated conditions for 24, 48, 72, and 96 h, 10 μL of CCK-8 reagent was placed in each well and incubated for 2 h. Absorbance at 450 nm was measured using a microplate reader (BioTek Instruments, Winooski, VT, USA, Synergy H1) to evaluate cell viability.

### Scratch wound healing assay

hMSCs were seeded in 6-well plates and cultured in complete medium (low-glucose DMEM, Thermo Fisher Scientific, Waltham, MA, USA, Cat. No. 11885-084) supplemented with 10% FBS (Gibco, Waltham, MA, USA, Cat. No. 10099-141) and 1% penicillin–streptomycin (Gibco, Waltham, MA, USA, Cat. No. 15140-122) until reaching 90% confluence. A linear scratch was formed with a sterile 200-µL pipette tip, and the wells were gently rinsed with PBS (Thermo Fisher Scientific, Waltham, MA, USA, Cat. No. 10010-023) to remove detached cells. Cells were then incubated in serum-free medium and imaged at 0 and 24 h via a light microscope (Leica Microsystems, Wetzlar, Germany, DM3000). The wound area was measured using ImageJ software, and the migration (healing) rate was calculated by comparing the residual wound area at 24 h to the initial area.

### Colony formation assay

MSCs were seeded into 6-well plates at a density of 500 cells per well in complete growth medium (low-glucose DMEM, Thermo Fisher Scientific, Waltham, MA, USA, Cat. No. 11885-084) supplemented with 10% FBS (Gibco, Waltham, MA, USA, Cat. No. 10099-141) and 1% penicillin–streptomycin (Gibco, Waltham, MA, USA, Cat. No. 15140-122). Cells were incubated at 37 °C with 5% CO_2_ for 10–14 days, with the medium refreshed every 3 days. At the end of the incubation period, colonies were immobilized with 4% paraformaldehyde (Thermo Fisher Scientific – Waltham, MA, USA, Cat. No. 28908) for 15 min and stained with 0.1% crystal violet solution (Abcam plc, Cambridge, United Kingdom, Cat. No. ab246784) for 30 min. Colonies containing more than 50 cells were counted under a light microscope (Leica Microsystems, Wetzlar, Germany, DM3000).

### Western blot analysis

MSCs were lysed using RIPA buffer (Thermo Fisher Scientific, Waltham, MA, USA, Cat. No. 89900) containing protease and phosphatase inhibitors (Cell Signaling Technology (CST), Danvers, MA, USA, Cat. No. 5872) on ice for 30 min. Total protein concentration was determined by BCA assay (Thermo Fisher Scientific, Waltham, MA, USA, Cat. No. 23225). Equal amounts of protein (20–30 µg) were separated by SDS-PAGE and transferred onto PVDF membranes (Millipore, Cat. No. IPVH00010). Membranes were blocked with 5% non-fat milk in TBST for 1 h at room temperature and incubated overnight at 4°C with primary antibodies against *PTGS2* (Cell Signaling Technology (CST), Danvers, MA, USA, Cat. No. 12282), *NR4A1* (Abcam plc, Cambridge, United Kingdom, Cat. No. ab13851), RANKL (Proteintech Group, Rosemont, IL, USA, Cat. No. 66610-1-Ig), OPG (Abcam plc, Cambridge, United Kingdom, Cat. No. ab73400), *RUNX2* (Cell Signaling Technology (CST), Danvers, MA, USA, Cat. No. 12556), and OCN (Abcam plc, Cambridge, United Kingdom, Cat. No. ab93876). After washing, membranes were incubated with HRP-conjugated secondary antibodies (Abcam plc, Cambridge, United Kingdom, Cat. No. ab97051) for 1 h at room temperature. Protein bands were visualized using enhanced chemiluminescence reagents (Thermo Fisher Scientific, Waltham, MA, USA, Cat. No. 34577) and imaged with a chemiluminescence detection system. β-Actin was used as the internal control. Densitometric analysis was performed using ImageJ software.

### Quantitative real-time PCR

Total RNA was extracted from MSCs using TRIzol reagent (Thermo Fisher Scientific, Waltham, MA, USA, Cat. No. 15596026) following the manufacturer’s instructions. RNA concentration and purity were determined using a NanoDrop spectrophotometer (Thermo Fisher Scientific, Waltham, MA, USA). cDNA was synthesized from 1 µg of total RNA using the PrimeScript RT Reagent Kit (TaKaRa Bio Inc., Kusatsu, Shiga, Japan, Cat. No. RR037A). Quantitative PCR was performed using SYBR Green Master Mix (Applied Biosystems, Foster City, CA, USA, Cat. No. 4309155) on a 7500 Real-Time PCR System (Applied Biosystems, Foster City, CA, USA). Gene-specific primers targeting *PTGS2*, *NR4A1*, RANKL, OPG, *RUNX2*, and OCN were synthesized by Sangon Biotech (Sangon Biotech Co., Ltd., Shanghai, China) (primer detailed in **Supplementary Table S1**). β-Actin was used as the internal reference. Relative expression levels were calculated using the 2^−ΔΔCt^ method.

### Dual-luciferase reporter assay

To evaluate the binding of *NR4A1* to the *PTGS2* promoter, dual-luciferase reporter assays were performed using the Dual-Luciferase^®^ Reporter Assay System (Promega Corporation, Madison, WI, USA, Cat. No. E1910). The wild-type *PTGS2* promoter sequence and three mutant constructs (each harboring a single site-directed mutation at predicted *NR4A1* binding motifs) were cloned into the pGL3-Basic luciferase reporter vector (Promega Corporation, Madison, WI, USA, Cat. No. E1751). All constructs were verified by Sanger sequencing. HEK293T cells were seeded into 24-well plates and co-transfected with 400 ng of reporter plasmid and 100 ng of pRL-TK Renilla luciferase vector (Promega Corporation, Madison, WI, USA, Cat. No. E2241) as an internal control via Lipofectamine 3000 (Thermo Fisher Scientific, Waltham, MA, USA, Cat. No. L3000008). Additionally, cells were co-transfected with 300 ng of *NR4A1* overexpression plasmid or an empty vector control. After 48 h, firefly and Renilla luciferase activities were measured sequentially using a microplate luminometer (Promega Corporation, Madison, WI, USA, GloMax^®^ Discover). Relative promoter activity was calculated as the ratio of firefly to Renilla luciferase activity.

### Chromatin immunoprecipitation-qPCR assay

Chromatin immunoprecipitation (ChIP) assays were performed using the SimpleChIP^®^ Enzymatic Chromatin IP Kit (Cell Signaling Technology, Danvers, MA, USA, Cat. No. 9003) following the manufacturer’s protocol. MSCs were treated with recombinant human *NR4A1* protein (rhNR4A1, R&D Systems, Minneapolis, MN, USA, Cat. No. 8456-NR), OPC compound at the indicated concentration, or anti-*NR4A1* neutralizing antibody (Abcam plc, Cambridge, United Kingdom, Cat. No. ab13851) for 24 h. Cells were crosslinked with 1% formaldehyde for 10 min and quenched with glycine. Chromatin was enzymatically digested and sonicated to yield DNA fragments of 150–900 bp. Immunoprecipitation was performed using anti-*NR4A1* antibody (Cell Signaling Technology (CST), Danvers, MA, USA, Cat. No. 13851) or normal IgG as a negative control. After reverse crosslinking, DNA was purified and analyzed by quantitative PCR using primers targeting the *PTGS2* promoter region. Enrichment was calculated relative to input DNA and normalized to IgG controls.

### ELISA for serum bone turnover markers

Serum levels of bone turnover markers, including C-terminal telopeptide of type I collagen (CTX) and procollagen type I N-terminal propeptide (PINP), were quantified leveraging commercial enzyme-linked immunosorbent assay (ELISA) kits in accordance with the manufacturers’ instructions. CTX was measured using the RatLaps ELISA kit (Immunodiagnostic Systems, Boldon, Tyne and Wear, United Kingdom, Cat. No. AC-06F1), and PINP was detected with the Human PINP ELISA kit (Cloud-Clone Corp., Wuhan, Hubei, China, Cat. No. SEA957Hu). Blood samples were collected, allowed to clot at room temperature, and centrifuged at 3,000×*g* for 10 min to isolate serum. Samples were stored at −80°C until analysis. Absorbance was measured at 450 nm using a microplate reader (BioTek Instruments, Winooski, VT, USA, Synergy H1), and concentrations were calculated based on standard curves.

### Micro-CT and histological analysis

Femurs were harvested and immersed in 4% paraformaldehyde (Thermo Fisher Scientific, Waltham, MA, USA, Cat. No. 28908) for 48 h, followed by micro-computed tomography (micro-CT) scanning using a SkyScan 1176 system (Bruker Corporation, Billerica, MA, USA) at a resolution of 9 μm. Three-dimensional reconstruction and quantitative analysis were performed using CTAn software (Bruker Corporation, Billerica, MA, USA) to evaluate trabecular bone parameters, including bone volume to tissue volume ratio (BV/TV), trabecular thickness (Tb.Th), and trabecular separation (Tb.Sp). For histological assessment, fixed samples were decalcified in 10% EDTA (pH 7.4) for 2–3 weeks, embedded in paraffin, and sectioned at 5 μm. Tartrate-resistant acid phosphatase (TRAP) staining was carried out with a TRAP staining kit (Sigma-Aldrich, St. Louis, MO, USA, Cat. No. 387A) according to the manufacturer’s protocol to identify osteoclasts. TRAP-positive multinucleated cells were counted under a light microscope (Leica Microsystems, Wetzlar, Germany, DM3000). All analyses were conducted in a blinded manner.

### Co-immunoprecipitation assay

To examine the interaction between *NR4A1* and *Runx2* in MSCs, co-immunoprecipitation (co-IP) assays were conducted using the Pierce™ Co-Immunoprecipitation Kit (Thermo Fisher Scientific, Waltham, MA, USA, Cat. No. 26149) according to the manufacturer’s instructions. Briefly, MSCs were lysed in IP lysis buffer supplemented with protease and phosphatase inhibitors (Cell Signaling Technology (CST), Danvers, MA, USA, Cat. No. 5872) on ice. Equal amounts of total protein (500–800 µg) were incubated overnight at 4°C with anti-*NR4A1* antibody (Abcam plc, Cambridge, United Kingdom, Cat. No. ab13851) or control IgG, followed by incubation with AminoLink™ Plus resin for 2 h at room temperature. Immunoprecipitated complexes were eluted, resolved by SDS-PAGE, and assessed by Western blotting via anti-*Runx2* antibody (Cell Signaling Technology (CST), Danvers, MA, USA, Cat. No. 12556). Input lysates and IgG controls were included to validate specificity. All experiments were independently repeated at least three times.

### Statistical analysis

R software was used for statistical analyses. Significance was evaluated using two-tailed *P*-values, with criteria defined as *P < 0.05, **P < 0.01, and ***P < 0.001.

## Results

### Mapping of the target sites and functional pathways of OPC based on network pharmacology

**Figure 1:**
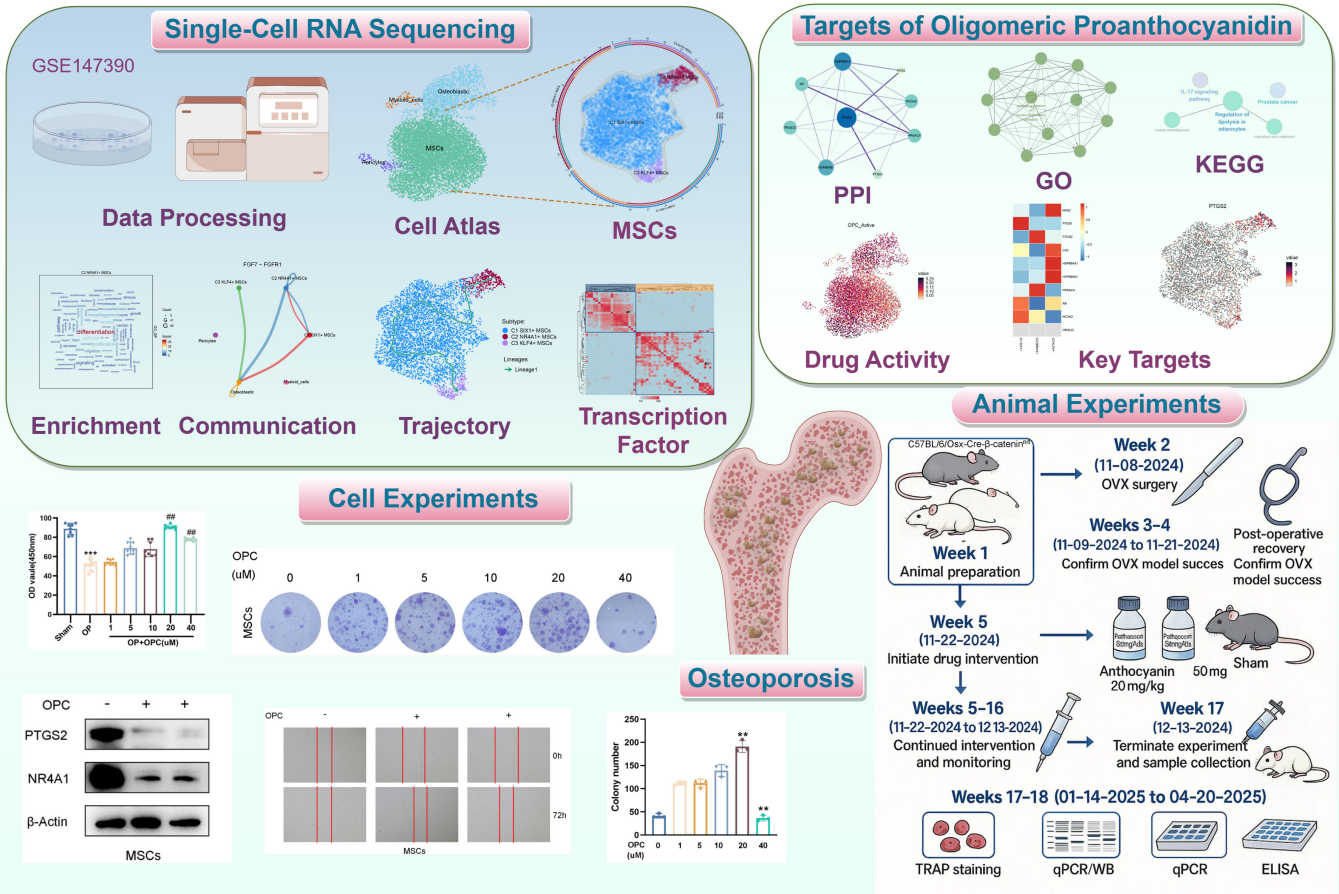
Flowchart. Schematic diagram of the analysis process based on network pharmacology and bioinformatics. **P < 0.01, ***P < 0.001 versus control; ^##^ *P* < 0.01 versus OP group.

Through a systematic network pharmacology analysis, we successfully identified 10 key target genes involved in the action of OPC, among which the target proteins corresponding to nine genes were able to form a stable PPI network (**Supplementary Figure S1A**). Notably, *PTGS2* was situated in the hub of the network of interactions, with the largest number of connection nodes and the highest connectivity. This suggested that it could have a significant regulatory function within the OPC action network. To provide further light on these target genes’ biological importance, we executed GO and KEGG pathway enrichment analysis (**Supplementary Figures S1B, C**), and the results were significantly enriched in the biological process of “positive regulation of nucleocytoplasmic transport” and the pathway of “regulation of lipolysis in adipocytes.” Based on this finding, we designed an integrated research plan including scRNA-seq, network pharmacology, cell experiments, and animal validation. **Figure 1** shows the entire workflow.

### Single-cell resolution reveals cellular heterogeneity in osteopenia and drug response features of OPC-targeted MSCs

**Figure 2:**
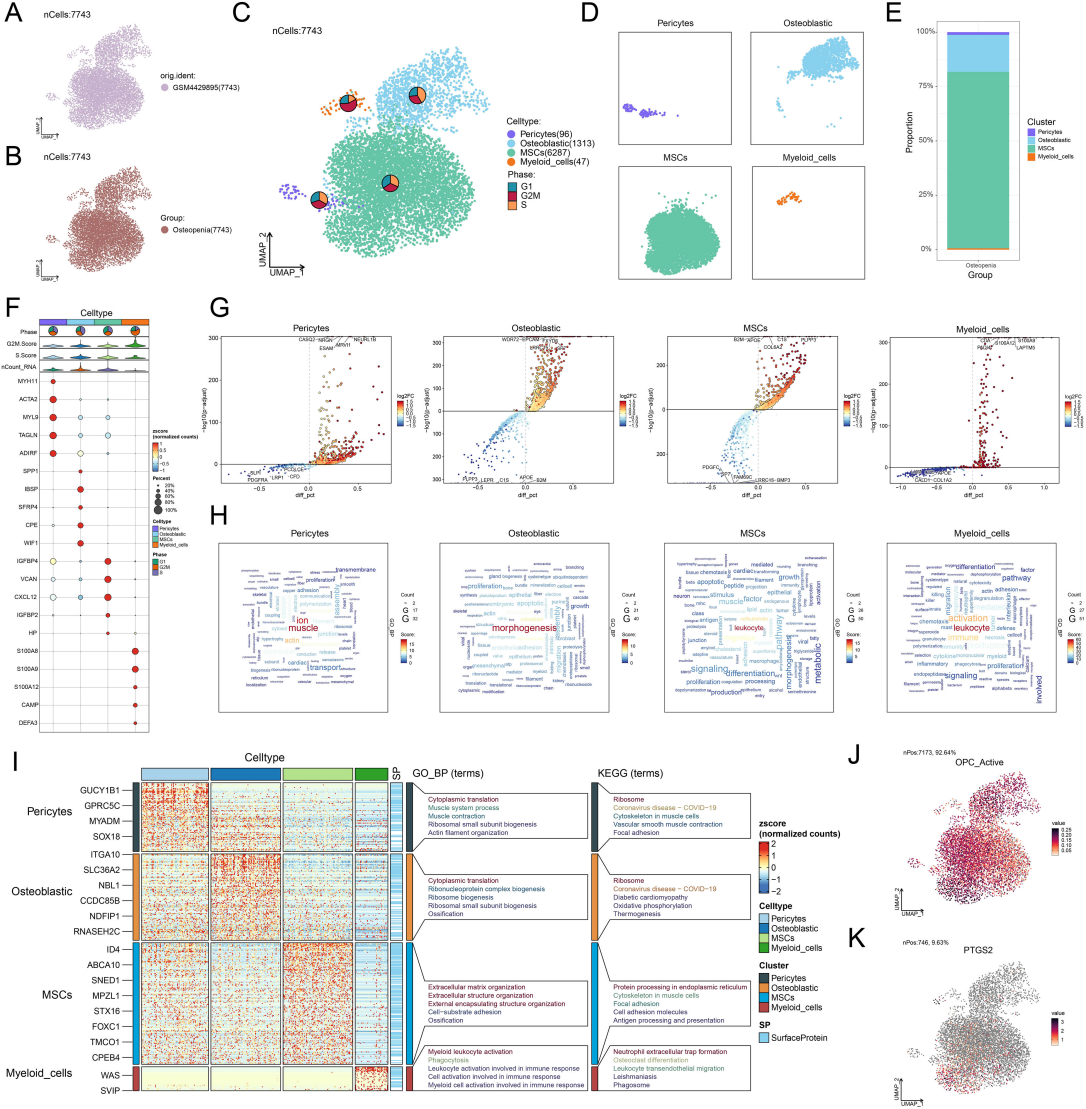
Single-cell atlas of osteopenia and cell-type-specific expression features of OPC target genes. **(A, B)** UMAP plots displayed the cellular distribution stratified by sample source and group (osteopenia). **(C)** The UMAP plot delineated the distribution of four cell types, accompanied by the embedded pie charts that further illustrated their proportional distribution across different cell cycle phases (G1, G2/M, S). **(D)** Four UMAP plots respectively depicted the distribution of each cell type. **(E)** The stacked bar plot showed the proportional distribution of four cell types within osteopenia. **(F)** The bubble plot depicted the expression levels of the top 5 marker genes across different cell types. The pie charts illustrated the proportional distribution of cell cycle phases, while the violin plots displayed the expression levels of G2/M. Score, S. Score, and nCount-RNA. The bubble size represented the percentage of gene expression, and the color indicated the *z*-score (normalized counts). **(G)** The volcano plots displayed significantly upregulated and downregulated genes in each cell type. **(H)** The word cloud graphs displayed the activity of different pathways in each cell type. **(I)** The heatmap showed the enrichment analysis results of the top 5 GO-BP and KEGG terms for four types of cells. **(J)** The UMAP plot presented the distribution of OPC active expression across all cell types. **(K)** The UMAP plot displayed the distribution of *PTGS2* across all cell types.

We used scRNA-seq technology to analyze osteopenia, successfully constructing a single-cell map of early OP (**Figures 2A, B**). After quality control screening, a total of 7,743 high-quality cells were acquired. Dimensionality reduction and clustering identified four major cell types, namely, pericytes, osteoblastic cells, MSCs, and myeloid cells, and embedded pie charts were used to display the variations in each cell type’s distribution at various phases of the cell cycle (**Figures 2C, D**). Further analysis revealed that compared with other cell types, the proportion of MSCs was higher in osteopenia (**Figure 2E**), hinting that they might be crucial to aberrant bone metabolism. We identified the top 5 marker genes for each cell type and genes that were highly upregulated and downregulated in order to better investigate the molecular traits of various cell types (**Figures 2F, G**). In **Figure 2H**, we found that pericytes were predominantly concentrated in muscle- and ion-related processes, while osteoblastic cells were involved in morphogenesis and osteoblast-related processes. Myeloid cells showed strong associations with leukocyte-related processes and activation, whereas MSCs were closely associated with leukocyte-related and cell-substrate processes. Moreover, MSCs were significantly enriched in a series of key biological processes, including extracellular matrix organization, extracellular structure organization, external encapsulating structure organization, cell-substrate adhesion, and ossification. In terms of key biological pathways, MSCs were mainly involved in protein processing in the endoplasmic reticulum, cytoskeleton in muscle cells, focal adhesion, cell adhesion molecules, and antigen processing and presentation (**Figure 2I**). Further analysis showed that OPC showed a high active expression level in MSCs (**Figure 2J**), and its key target gene *PTGS2* was also highly expressed in MSCs (**Figure 2K**), further supporting its important role in bone metabolism regulation. Based on this, we believe that the functional state and heterogeneity changes of MSCs may be an important pathological basis for the progression from osteopenia to OP.

### Single-cell profiling revealed the heterogeneity of MSC subtypes and their functional characteristics in osteopenia

**Figure 3:**
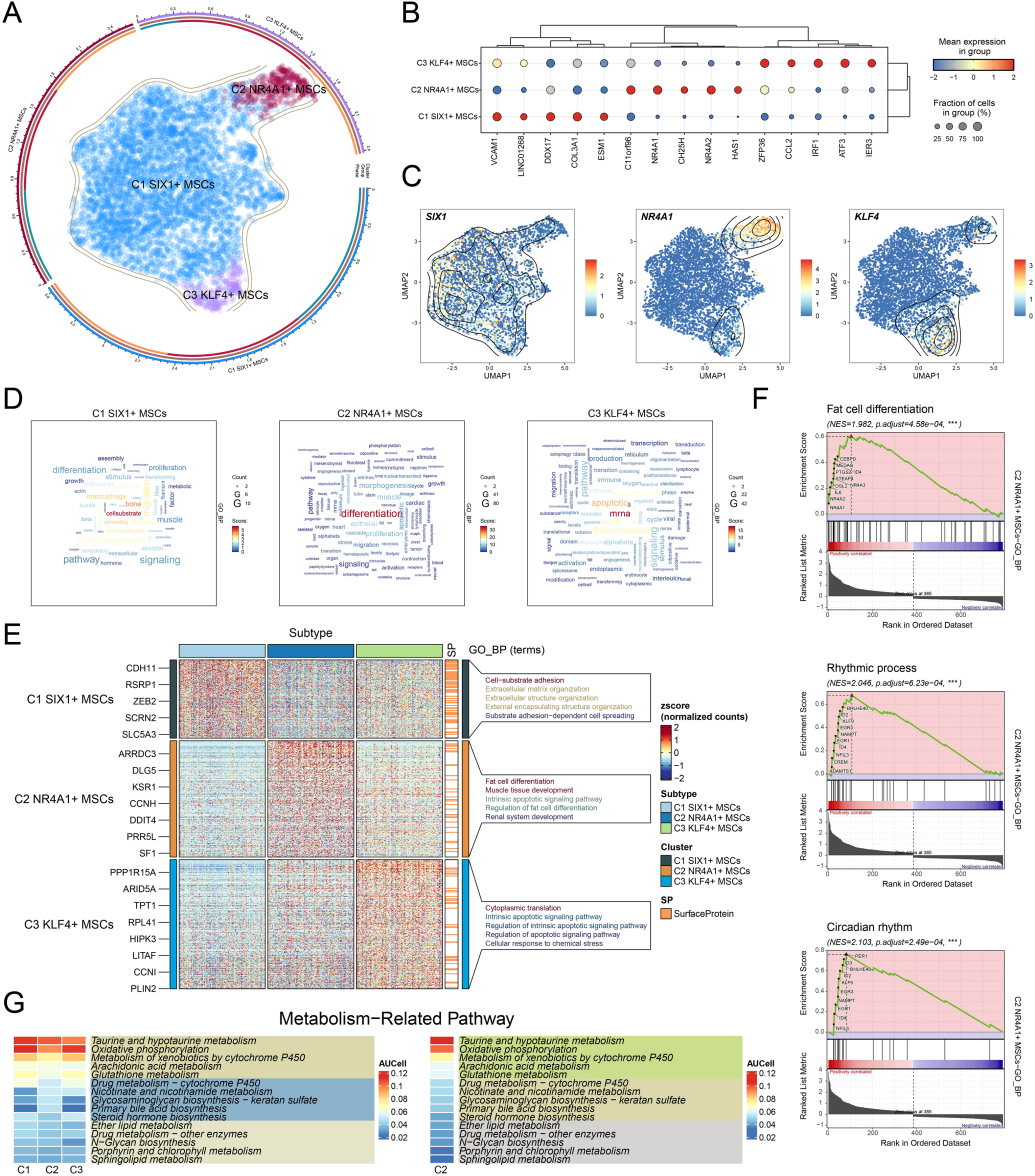
Single-cell atlas of MSC subtypes in osteopenia. **(A)** Circular plot illustrated the clustering of three MSC subtypes in osteopenia, with contour curves depicting the distribution of each subtype. The outer, middle, and inner axes represented the log-scaled clusters, groups, and cell cycle phases of each subtype, respectively. **(B)** The bubble plot displayed the mean expression levels of the top 5 DEGs in each MSC subtype. The bubble size corresponded to the percentage of gene expression, while the color represented normalized data. **(C)** UMAP plots illustrated the distribution of signature genes across three MSC subtypes. Contour density lines were overlaid to highlight regions with higher gene expression intensity. **(D)** The word cloud graphs presented the activity of different pathways in each MSC subtype. **(E)** The heatmap demonstrated the enrichment analysis results of the top 5 GO-BP terms for the three types of cells. **(F)** The GSEA enrichment analysis revealed the GO-BP terms related to the DEGs in C2 *NR4A1*+ MSCs. ***P < 0.001. **(G)** The heatmap revealed the metabolism-related pathways enriched in different MSC subtypes and C4 *NR4A1*+ MSCs.

To further analyze the diversity of MSCs in osteopenia and their potential functional differentiation, we conducted a detailed subtype analysis and identified three MSC subtypes with different molecular characteristics and functional states: C1 *SIX1*+ MSCs, C2 *NR4A1*+ MSCs, and C3 *KLF4*+ MSCs (**Figure 3A**). We identified the five most DEGs with the highest mean expression levels in each MSC subtype (**Figure 3B**) and validated the uniqueness of each MSC subtype through signature gene distribution analysis (**Figure 3C**). Pathway activity analysis showed that these subtypes exhibited distinct functional differentiation: C1 *SIX1*+ MSCs were primarily involved in cell-substrate and bone-related processes, C2 *NR4A1*+ MSCs were closely associated with differentiation and leukocyte-related functions, while C3 *KLF4*+ MSCs participated in mRNA regulation and apoptotic processes (**Figure 3D**). Further GO-BP enrichment analysis (**Figure 3E**) supported this functional distinction. C1 *SIX1*+ MSCs were mainly enriched in cell-substrate adhesion, extracellular matrix organization, extracellular structure organization, external encapsulating structure organization, and substrate adhesion-dependent cell spreading. C2 *NR4A1*+ MSCs were significantly involved in fat cell differentiation, muscle tissue development, intrinsic apoptotic signaling pathway, regulation of fat cell differentiation, and renal system development. C3 *KLF4*+ MSCs were closely related to cytoplasmic translation, intrinsic apoptotic signaling pathway, regulation of intrinsic apoptotic signaling pathway, regulation of apoptotic signaling pathway, and cellular response to chemical stress. Based on GSEA analysis, we found that fat cell differentiation, rhythmic process, and circadian rhythm were significantly upregulated in C2 *NR4A1*+ MSCs (**Figure 3F**). Moreover, C2 *NR4A1*+ MSCs showed significant enrichment in multiple metabolic pathways, including taurine and hypotaurine metabolism, oxidative phosphorylation, metabolism of xenobiotics by cytochrome P450, arachidonic acid metabolism, and glutathione metabolism (**Figure 3G**). These results showed that C2 *NR4A1*+ MSCs may be essential for regulatory and metabolic integration during the early stage of osteopenia, laying the foundation for further investigation into their role in disease progression.

### Analysis of differentiation characteristics of three MSC subtypes

**Figure 4:**
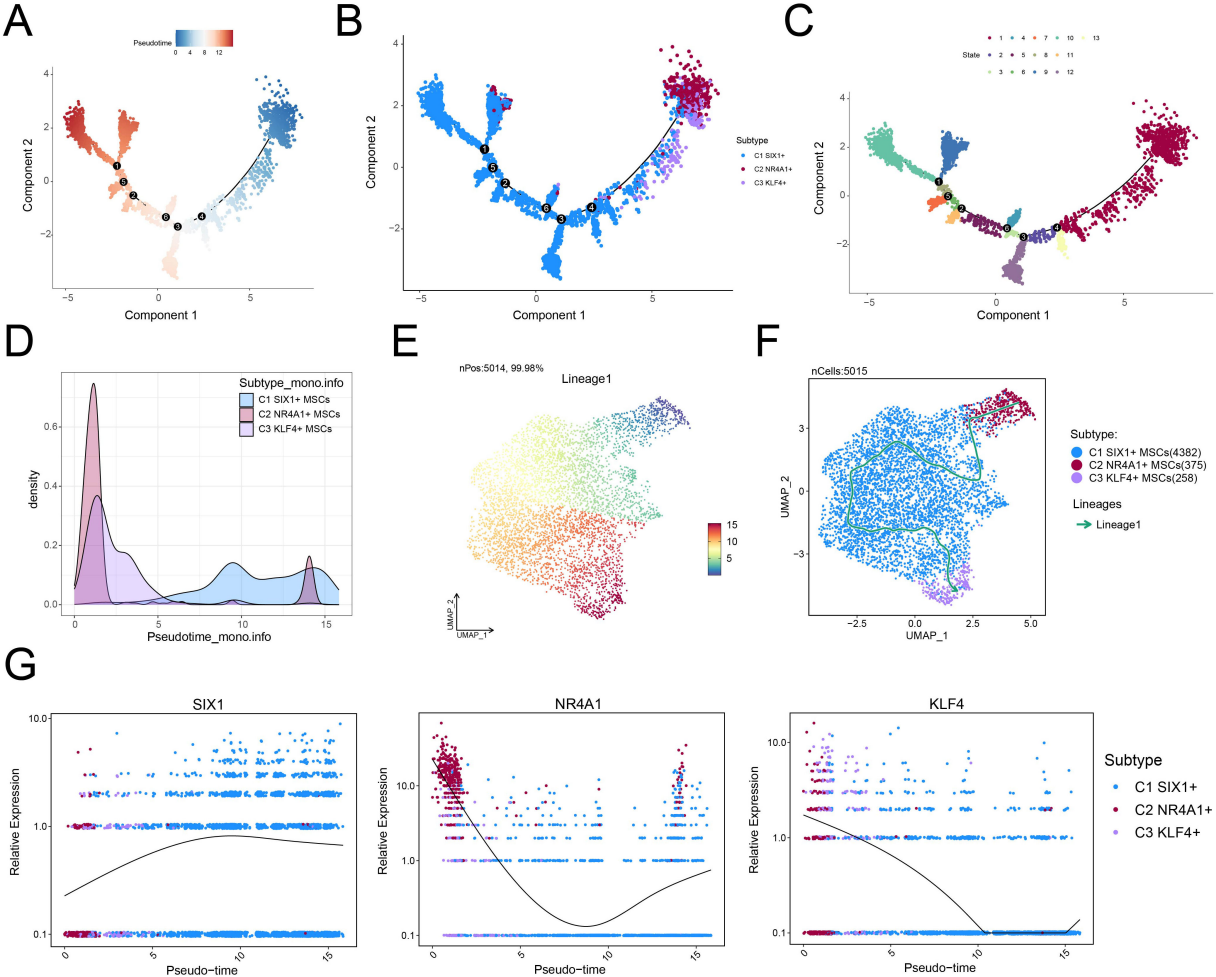
Single-cell profiling revealed the differentiation trajectory of MSCs. **(A)** Monocle analysis revealed the developmental trajectory of MSCs. **(B)** Cells were color-coded by pseudotime to visualize the trajectory distribution of three MSC subtypes. **(C)** The cell development trajectory was divided into 13 time states based on the pseudotemporal sequence. **(D)** Ridge plots demonstrated the pseudotime-dependent dynamic changes across MSC subtypes. **(E)** The UMAP plot illustrated the distribution of lineage 1 along the inferred pseudotemporal sequence. **(F)** UMAP plot revealed differentiation trajectories and lineages of three MSC subtypes through Slingshot analysis. **(G)** The dynamic trend plots showed the relative expression of signature genes for each MSC subtype across pseudotime.

To elucidate the differentiation dynamics of MSC subtypes under osteopenia conditions, we performed pseudotime trajectory analysis using both Monocle and Slingshot. The developmental trajectory of MSCs originated in the upper right quadrant and extended along the main path toward the left side (**Figures 4A–C**). The results showed that C2 *NR4A1*+ MSCs were chiefly situated at the inception of the developmental trajectory (**Figures 4C, D**) and exhibited a differentiation path of C2→C1→C3 along the major lineage (lineage 1) (**Figures 4E, F**). This trajectory suggested that C2 *NR4A1*+ MSCs might represent the initial state of MSC differentiation, with C1 *SIX1*+ MSCs as an intermediate functional transition stage and C3 *KLF4*+ MSCs as a more terminal state. Signature gene expression analysis further confirmed the high expression profile of C2 *NR4A1*+ MSCs at the early stage (**Figure 4G**), indicating their potential pivotal role in directing MSC differentiation and functional shift. Therefore, focusing on C2 *NR4A1*+ MSCs not only helped to deepen our understanding of MSC fate determination but also provided clues for identifying critical time windows to intervene in the progression of osteopenia.

### Intercellular communication network within the osteopenic bone marrow niche

**Figure 5:**
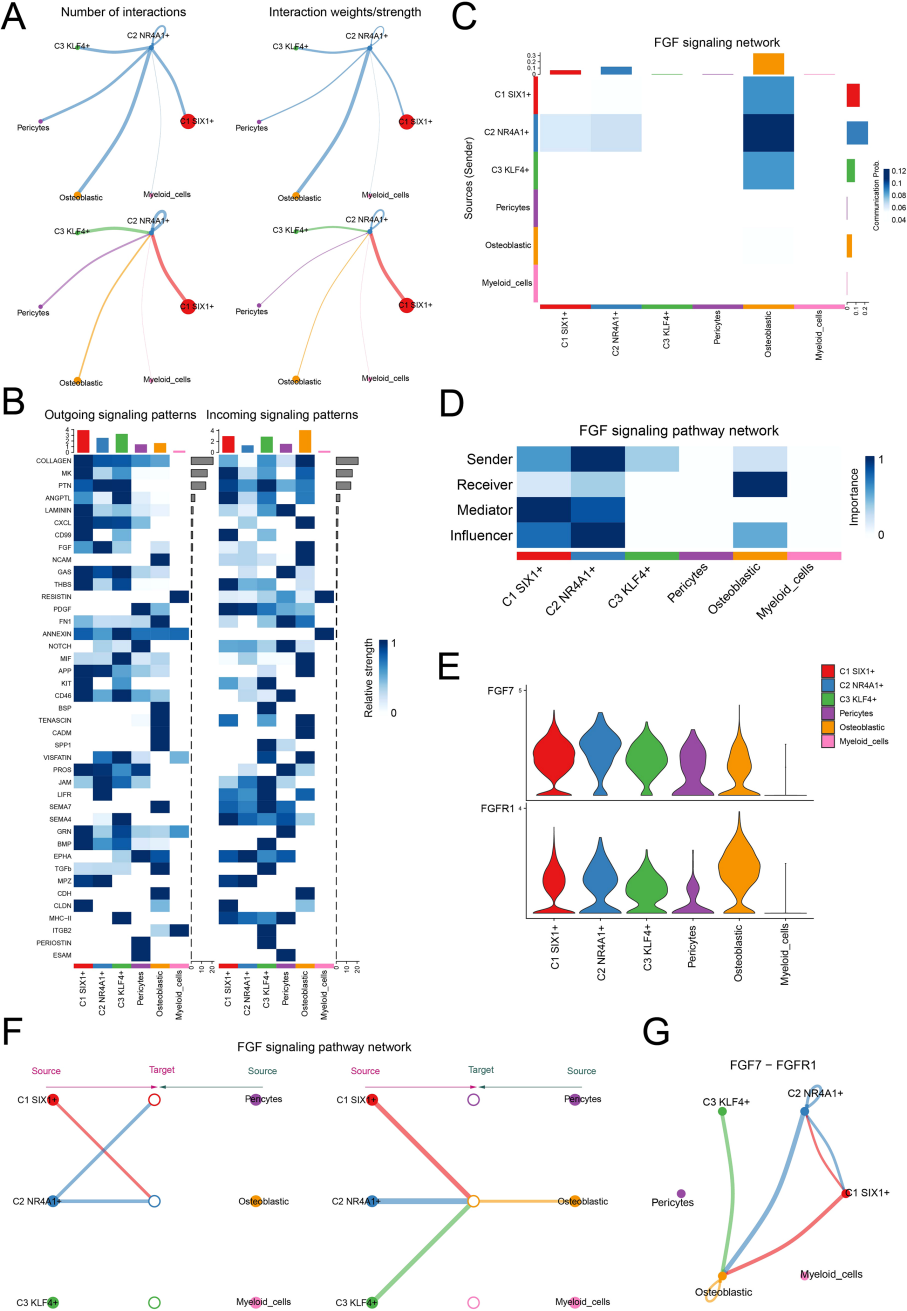
Cell–cell communication atlas. **(A)** The circle plot displayed the number of interactions (left) and interaction strength (right) between C2 *NR4A1*+MSCs, as sources and targets, and other cells. **(B)** The heatmap described the relative strength of various signaling pathways in the outgoing and incoming signaling patterns of three MSC subtypes and three other cell types. **(C)** The heatmap quantified the communication probabilities between three MSC subtypes and three other cell types within the FGF signaling network. **(D)** The heatmap depicted the centrality scores of the FGF signaling pathway network. **(E)** The violin plot visualized the expression levels of key ligand and receptor in the FGF signaling pathway across three MSC subtypes and three other cell types. **(F)** The hierarchical plot depicted the interactions between three MSC subtypes and three other cell types within the FGF signaling pathway network. **(G)** The circle plot described the interactions between three MSC subtypes and three other cell types within the FGF7–FGFR1 signaling network.

Based on the central role of C2 *NR4A1*+ MSC in the early stages of MSC differentiation, we further investigated their intercellular communication within the osteopenia-associated microenvironment. Through cell interaction analysis, we found significant signal exchange between C2 *NR4A1*+ MSC and osteoblastic cells (**Figure 5A**). Analysis of outgoing and incoming signaling patterns revealed that the relative strength of the fibroblast growth factor (FGF) signaling pathway was particularly prominent between these two cell types (**Figure 5B**). Systematic analysis showed that under the FGF signaling network, the communication probability between C2 *NR4A1*+ MSC and osteoblastic cells was markedly increased (**Figure 5C**). Moreover, these two cell types exhibited a clear functional division within the signaling network: C2 *NR4A1*+ MSCs primarily acted as signal senders, mediators, and influencers, while osteoblastic cells mainly functioned as receivers (**Figure 5D**). By analyzing the expression of key ligand and receptor, we ascertained that FGF7 and FGFR1 were strikingly upregulated in both C2 *NR4A1*+ MSCs and osteoblastic cells (**Figure 5E**), providing a molecular basis for explaining the specific communication between the two. Integrated analysis of the FGF signaling network revealed that C2 *NR4A1*+ MSCs and osteoblastic cells formed a specific signaling connection (**Figure 5F**), with the communication network established through the FGF7–FGFR1 signaling axis displaying significant interactive characteristics (**Figure 5G**). These findings not only highlighted the critical role of the C2 *NR4A1*+ MSCs in regulating bone metabolic balance but also revealed a highly specific regulatory network within the bone marrow microenvironment, offering potential targets for future therapeutic intervention.

### Transcriptional regulatory features of MSC subtypes

**Figure 6:**
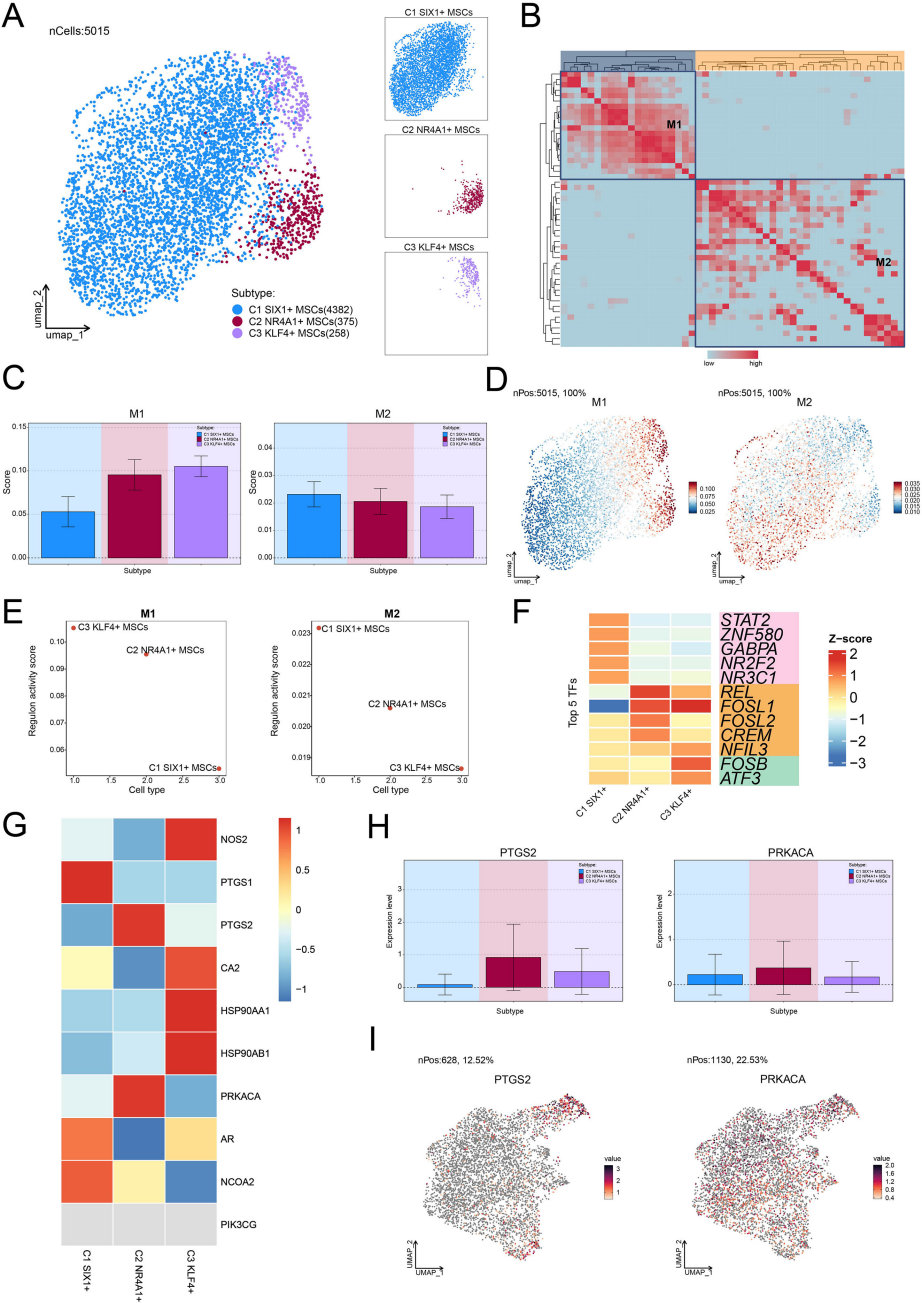
Transcriptional regulatory networks and OPC response characteristics in MSC subtypes. **(A)** The UMAP plot on the left visualized the clustering distribution of three MSC subtypes based on TF activation levels, while the UMAP plots on the right highlighted each MSC subtype’s distribution separately. **(B)** The heatmap displayed the two regulatory modules determined based on pySCENIC regulatory rules and AUCell similarity scores. **(C)** Bar plots provided the AUC scores of TFs across different MSC subtypes within the two regulatory modules. **(D)** UMAP plots depicted the differential expression distribution of TFs within the two regulatory modules. **(E)** Scatter plots provided the ranking of regulon activity score for different MSC subtypes within the two regulatory modules. **(F)** The heatmap demonstrated the top 5 TFs of each MSC subtype. **(G)** The heatmap displayed the expression of 10 target genes of OPC across different MSC subtypes. **(H, I)** Bar plots and UMAP plots visualized the expression levels and distribution of two target genes of OPC in C2 *NR4A1*+ MSCs, respectively.

To further explore the upstream regulatory mechanisms underlying the function of C2 *NR4A1*+ MSCs, we systematically analyzed their transcriptional regulatory network. This study first revealed a subtype-specific distribution pattern based on the activation levels of TFs (**Figure 6A**) and identified two functionally distinct regulatory modules (M1 and M2) using pySCENIC regulatory rules and AUCell similarity scores (**Figure 6B**). We observed that C2 *NR4A1*+ MSC displayed a marked regulatory advantage in M1 compared with M2 (**Figures 6C–E**). Specifically, C2 *NR4A1*+ MSC in M1 showed higher AUC scores, increased TF expression, and enhanced regulon activity relative to those in M2. Subsequently, we identified the top 5 TFs of C2 *NR4A1*+ MSC—REL, FOSL1, FOSL2, CREM, and NFIL3—highlighting their unique epigenetic regulatory landscape (**Figure 6F**).

### Identification of key target genes for OPC treatment in OP

To further elucidate the role of OPC, we focused on investigating the response characteristics of different MSC subtypes to OPC. Through a systematic analysis of the expression profiles of the OPC target genes, we noticed that *PTGS2* and *PRKACA* were significantly expressed in C2 *NR4A1*+ MSC (**Figure 6G**). UMAP dimensionality reduction and quantitative expression comparisons further confirmed this discovery (**Figures 6H, I**). These findings suggested that OPC may regulate the bone marrow microenvironment by acting on C2 *NR4A1*+ MSC, thereby participating in the pathological process of osteopenia.

### OPC suppresses *PTGS2* expression in MSCs via inhibition of *NR4A1* transcriptional activity

**Figure 7:**
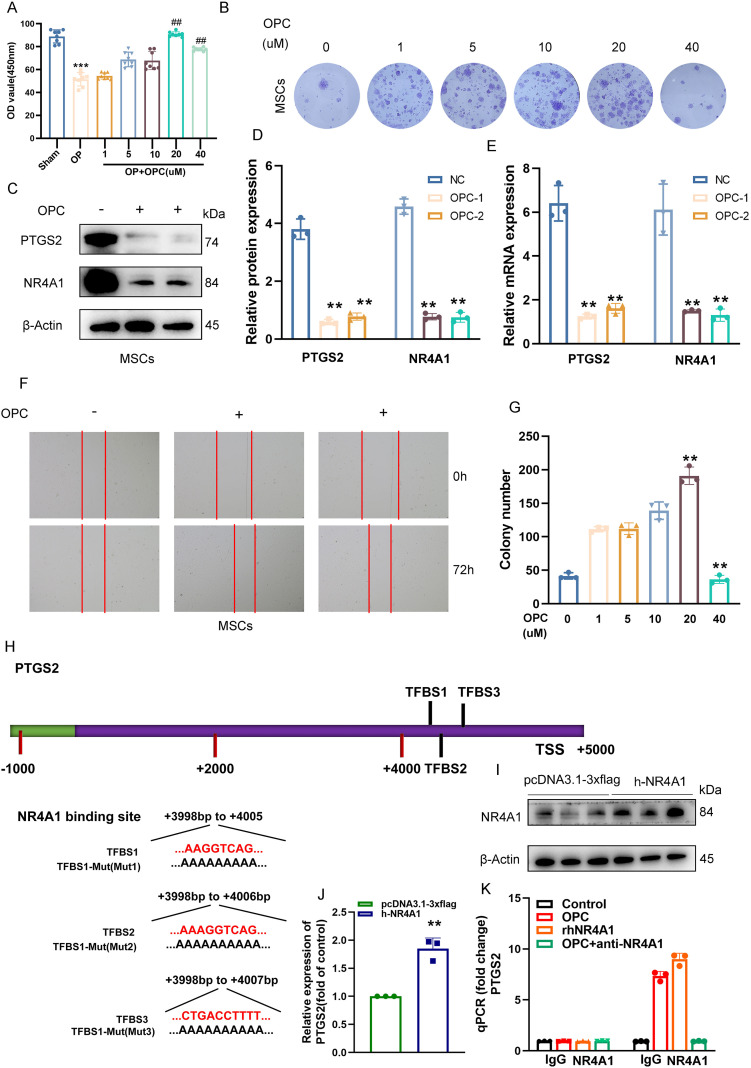
OPC suppresses *PTGS2* expression in MSCs via inhibition of *NR4A1* transcriptional activity. **(A)** The CCK-8 assay showing the effects of various concentrations of OPC (1–40 μM) on MSC viability. **(B)** Representative images of crystal violet-stained colonies formed by MSCs treated with increasing concentrations of OPC. **(C, D)** Western blot and quantification showing reduced protein levels of *PTGS2* and *NR4A1* upon OPC treatment. **(E)** RT-qPCR results confirming the downregulation of *PTGS2* and *NR4A1* mRNA in OPC-treated MSCs. **(F)** Representative images of wound healing assays demonstrating enhanced migratory ability of MSCs upon OPC exposure. **(G)** Quantification of colony number showing a peak at 20 μM OPC, followed by a decline at 40 μM. **(H)** Schematic representation of the *PTGS2* promoter indicating three *NR4A1*-binding sites (TFBS1–3) and their mutated sequences. **(I)** Western blot confirming *NR4A1* overexpression in MSCs. **(J)** RT-qPCR showing increased *PTGS2* transcription upon *NR4A1* overexpression (*P* < 0.01). **(K)** ChIP-qPCR demonstrating *NR4A1* binding to the *PTGS2* promoter, which is reduced by OPC or anti-*NR4A1* treatment. Data are presented as mean ± SEM. **P < 0.01, ***P < 0.001 versus control; ^##^ *P* < 0.01 versus the OP group.

To evaluate the effect of OPC on MSCs, we first assessed cell viability via the CCK-8 assay. As shown in **Figure 7A**, OPC treatment significantly enhanced MSC viability in a dose-dependent manner, with the most notable increase observed at 10–20 μM, while 40 μM caused a slight reduction. Colony formation assays further confirmed that OPC promoted MSC proliferation, with increased colony numbers up to 20 μM (**Figures 7B, G**).

Western blot analysis revealed that OPC treatment markedly suppressed the expression of *PTGS2* and *NR4A1* proteins in MSCs (**Figures 7C, D**), which was further validated at the mRNA level by RT-qPCR (**Figure 7E**). Scratch wound healing assays demonstrated that OPC enhanced MSC migration at appropriate doses (**Figure 7F**), suggesting a pro-regenerative effect.

To determine the underlying regulatory mechanism, we identified three *NR4A1* binding sites (TFBS1–3) located in the distal promoter region of *PTGS2* (**Figure 7H**). Site-directed mutagenesis was performed to generate mutants for each predicted binding site.

Overexpression of *NR4A1* was confirmed by Western blotting in MSCs (**Figure 7I**), and RT-qPCR showed that *NR4A1* upregulation significantly increased *PTGS2* mRNA levels (**Figure 7J**). Furthermore, ChIP-qPCR revealed that *NR4A1* directly binds to the *PTGS2* promoter, while OPC treatment or anti-*NR4A1* antibody abrogated this interaction (**Figure 7K**), indicating that OPC inhibits *PTGS2* expression by interfering with *NR4A1*-mediated transcriptional regulation.

### OPC attenuates OVX-induced bone loss via a β-catenin-dependent *NR4A1*–*Runx2* signaling axis

**Figure 8:**
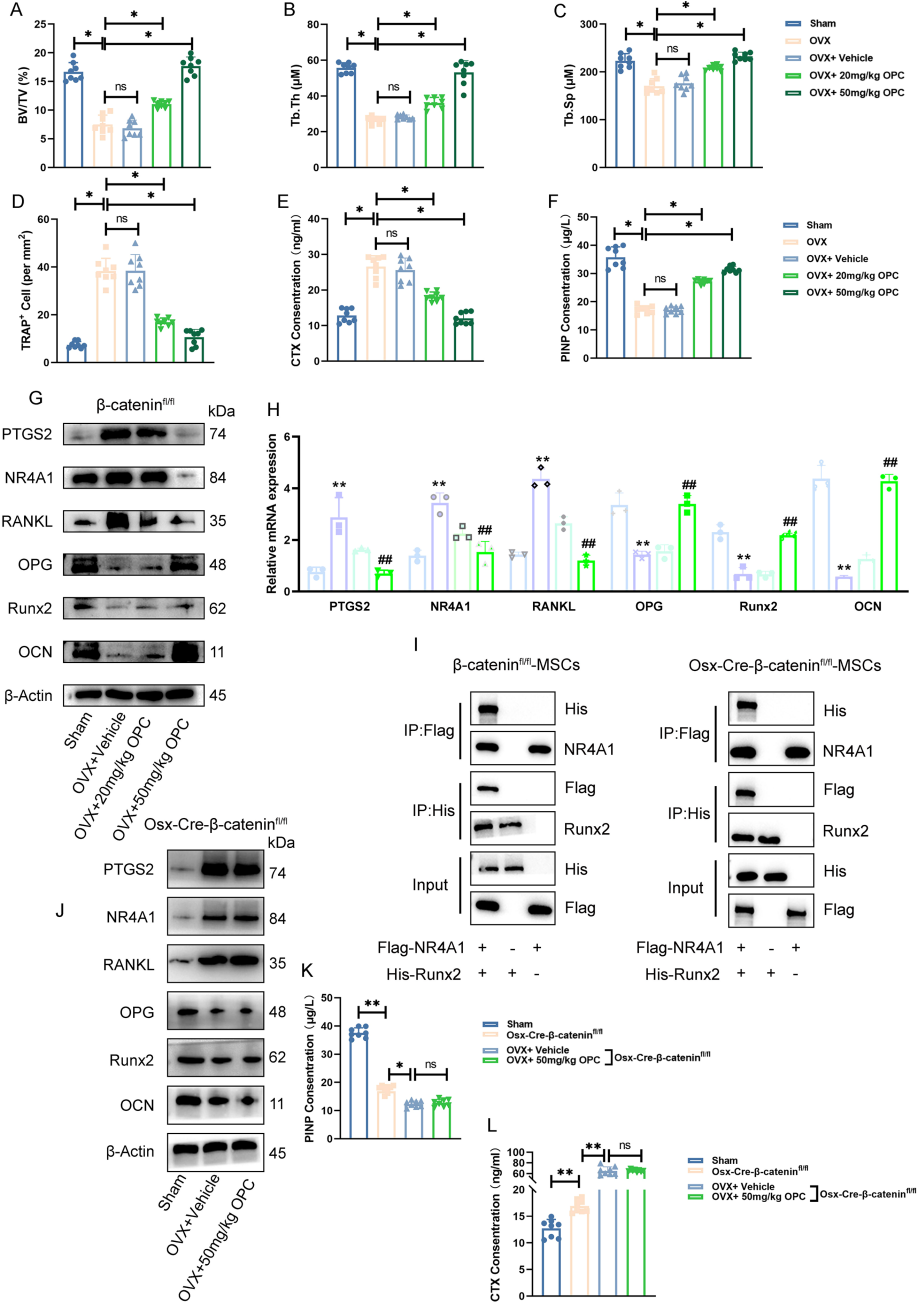
OPC attenuates OVX-induced bone loss via a β-catenin-dependent *NR4A1*–*Runx2* signaling axis. **(A–C)** Micro-CT analysis showing bone volume/total volume (BV/TV), trabecular thickness (Tb.Th), and trabecular separation (Tb.Sp) in femurs of sham, OVX, and OPC-treated mice. **(D)** Quantification of TRAP^+^ osteoclast numbers. **(E, F)** Serum CTX and PINP levels assessed by ELISA. **(G)** Western blot of *PTGS2*, *NR4A1*, RANKL, OPG, *Runx2*, and OCN in femurs. **(H)** RT-qPCR analysis of corresponding gene expression. **(I)** Co-immunoprecipitation showing interaction between Flag-*NR4A1* and His-*Runx2* in β-catenin^fl/fl^ and Osx-Cre-β-catenin^fl/fl^ MSCs. **(J)** Protein expression of target genes in femurs from β-catenin-deficient mice treated with OPC. **(K, L)** ELISA analysis of serum PINP and CTX levels in β-catenin^fl/fl^ versus Osx-Cre-β-catenin^fl/fl^ mice. Data are shown as mean ± SEM. *P < 0.05, **P < 0.01 versus indicated groups; ^##^ *P* < 0.01 versus OVX + vehicle.

To investigate the therapeutic effect of OPC in OP, an OVX-induced bone loss model was employed. Micro-CT uncovered that OPC significantly restored bone volume/total volume (BV/TV), trabecular thickness (Tb.Th), and spacing (Tb.Sp) compared to OVX controls (**Figures 8A–C**). Histological TRAP staining manifested a conspicuous reduction in osteoclast number in OPC-treated mice (**Figure 8D**), while biochemical markers of bone turnover, including CTX and PINP, were favorably modulated (**Figures 8E, F**).

Western blotting demonstrated that OVX increased *PTGS2*, *NR4A1*, and RANKL expression, while decreasing osteogenic markers such as OPG, *Runx2*, and OCN (**Figure 8G**). OPC treatment reversed these molecular changes in a dose-dependent manner. RT-qPCR data further confirmed the regulatory effects of OPC on these genes (**Figure 8H**), suggesting its potential to modulate both osteoclastogenic and osteogenic signaling.

To explore the mechanistic pathway, we performed co-immunoprecipitation in β-catenin^fl/fl^ and Osx-Cre-β-catenin^fl/fl^ MSCs. The interaction between *NR4A1* and *Runx2* was confirmed in wild-type MSCs but was markedly reduced in β-catenin-deficient cells (**Figure 8I**), indicating that β-catenin is required for *NR4A1*–*Runx2* complex formation.

In Osx-Cre-β-catenin^fl/fl^ mice, OPC failed to suppress *PTGS2*/*NR4A1*/RANKL or restore OPG/*Runx2*/OCN expression (**Figure 8J**). Furthermore, serum PINP and CTX levels showed no significant improvement with OPC in β-catenin-deficient mice (**Figures 8K, L**), confirming that the bone-protective effects of OPC are β-catenin dependent.

## Discussion

OP is a common metabolic bone disorder (15). Its pathological core lies in the persistent imbalance of bone homeostasis, manifested as the long-term dominance of osteoclast-mediated bone resorption and relatively insufficient osteoblast-driven bone formation capacity (4, 5). This imbalance is not only the result of local bone metabolic abnormalities but also a comprehensive manifestation of the systemic dysregulation of the bone microenvironment, involving multiple levels of interactions such as cellular heterogeneity, immune inflammation, and oxidative stress (1, 13, 56). Current clinical therapies, such as anti-resorption drugs and anabolic agents, can partially alleviate symptoms, but due to their single-target mechanisms, significant side effects during long-term use, and the difficulty in truly promoting bone structure regeneration, their therapeutic efficacy has obvious limitations.

OPC, as a class of natural plant pigments, is significantly different from traditional anti-OP drugs due to its outstanding safety features. It demonstrates unique advantages in the long-term intervention of chronic bone metabolic diseases. Its therapeutic potential lies in a multidimensional mechanism of action involving “inhibition of bone resorption, encouragement of bone formation, anti-inflammatory, and antioxidant effects” (16, 25). Previous studies have shown that various natural small molecules can improve bone homeostasis imbalance through different molecular pathways. For instance, luteolin, a natural flavonoid, can upregulate the expression of osteogenic-related proteins such as *Runx2* by activating the PI3K/Akt pathway, thereby reducing bone loss (57). Gastrodin mainly promotes osteogenic differentiation by stimulating the Wnt/β-catenin signaling and enhancing *Runx2* expression (58). Baicalein improves bone quality by upregulating *SIRT1*, *AR*, and *ESR1* and downregulating *PTGS2* expression (59). The small molecule drug metformin can restore *NR4A1*-mediated autophagic flux, showing significant protective effects against postmenopausal OP (60). Compared with these mechanisms mainly based on single pathways or local links, the action of OPC presents more comprehensive and bidirectional regulatory characteristics, highlighting its uniqueness and potential advantages in the treatment of OP.

To deeply reveal the specific mechanism of OPC in treating OP, we employed scRNA-seq to systematically study the bone microenvironment of OP. Osteopenia (bone mineral density *T*-score between −1.0 and −2.49), as a precursor stage to OP (*T*-score ≤−2.5) (61, 62), affects over 60% of individuals over the age of 60, with a prevalence approximately three times higher than that of OP (63). Notably, although osteopenia does not meet the diagnostic criteria for OP, more than half of osteoporotic fractures actually occur in individuals with osteopenia, while patients with OP account for only 20%–30% of the total fracture burden (63, 64). Osteopenia represents a critical window of intervention due to its rapid rate of bone loss, particularly in early postmenopausal stages or following the cessation of hormone therapy. Therefore, to elucidate the potential mechanisms by which OPCs treat OP, we performed a systematic analysis of the bone microenvironment in osteopenia samples using scRNA-seq technology.

From the data acquisition, we identified four major cell types in osteopenia. Among them, MSCs showed a significantly increased proportion in osteopenia, suggesting that they may play a key driving role in the imbalance of bone homeostasis. As the core stromal cell population in the bone marrow microenvironment, MSCs possess unique self-renewal capabilities and multidirectional differentiation potential and can differentiate into various lineages such as osteoblasts/bone cells, chondrocytes, and adipocytes, playing an irreplaceable role in maintaining the homeostasis of bone tissue (7, 56, 65). Particularly noteworthy is that as the direct source of osteoblasts, the balance of differentiation of MSCs into osteoblasts and adipocytes directly regulates bone metabolism (7, 66). Their differentiation imbalance (such as insufficient osteogenesis or excessive adipogenesis) is a major driver of the formation and progression of bone metabolic diseases such as OP (67). Therefore, MSCs are not only key regulators for understanding the mechanisms of bone metabolism but also represent potential therapeutic targets for OP.

This study further evaluated the pharmacological response characteristics of OPCs targeting MSCs. The results showed that OPCs exhibited relatively high activity in MSCs, suggesting that MSCs may serve as the primary targets through which OPCs exert their effects on OP. Additionally, the key target gene *PTGS2* was also highly expressed in MSCs, indicating that it may be an important molecular mediator of the bone-protective effects of OPCs. These observations provide a new molecular basis for understanding how OPCs intervene in OP by modulating MSC function.

In-depth analysis revealed that MSCs can be divided into three functionally heterogeneous subtypes. Among them, C2 *NR4A1*+ MSCs exhibited unique metabolic and rhythmic regulatory characteristics. This subtype was enriched in fat cell differentiation and circadian rhythm pathways. Previous studies showed that lipid metabolic abnormalities, including enhanced fat cell differentiation, regulated the functions of osteoblasts and osteoclasts (68, 69). In addition, circadian rhythm also influenced bone remodeling and bone metabolism, thereby accelerating osteopenia and the progression of OP (70, 71). At the same time, it has advantages in oxidative phosphorylation and glutathione metabolism, indicating its role as a metabolic hub coordinating energy metabolism and antioxidant defense, maintaining the balance between osteoblast and osteoclast (72–74). Differentiation trajectory analysis also revealed that C2 *NR4A1*+ MSCs are primarily located at the early stage of differentiation and participate in early differentiation regulation through specific lineage trajectories, presenting a new perspective on the mechanisms of MSC differentiation in OP.

In the analysis of the cellular communication network within the bone marrow microenvironment, we further found that under the FGF signaling pathway network, C2 *NR4A1*+ MSCs and osteoblasts exhibited significantly elevated signaling intensity and communication probability. The FGF signaling pathway is one of the core mechanisms regulating bone development and metabolism, playing a crucial role in maintaining the homeostasis of bone tissue (the dynamic balance between bone formation and bone resorption) (75). Meanwhile, the FGF signaling pathway regulates bone formation and resorption through the collaboration of multiple genes and is significantly associated with the pathogenesis of bone diseases such as OP (76). Subsequently, the transcriptional regulatory network of C4 *NR4A1*+ MSCs was thoroughly examined, and the top 5 TFs (REL, FOSL1, FOSL2, CREM, and NFIL3) with the highest activity in this subtype were found. This finding not only reveals the regulatory hub driving the unique functions of C2 *NR4A1*+ MSCs but also further supports their potential as key cellular targets for OP therapy. Notably, we discovered that OPCs can specifically target and act on C2 *NR4A1*+ MSCs, with this specificity being particularly evident at two key OPC targets: *PTGS2* and *PRKACA*.

*PTGS2* (Prostaglandin Endoperoxide Synthase 2/COX-2) is the rate-limiting enzyme in prostaglandin production and participates in physiological processes (like embryonic development and transportation of the reproductive system), as well as a key regulatory node in the pathogenesis of OP (77). Current research has revealed that *PTGS2* drives bone homeostasis imbalance through two pathways: on the one hand, its high expression promotes the release of inflammatory mediators (such as PGE2), activates osteoclast differentiation, and inhibits osteogenesis (78, 79); on the other hand, as a key gene in ferroptosis, *PTGS2* directly induces ferroptosis in BMSCs, leading to accumulation of ROS, iron overload, and mitochondrial damage, thereby blocking osteogenic differentiation and mineralization (80). Targeted therapy against *PTGS2* has become a new strategy for the intervention of OP. Natural compounds such as curcumin inhibit *PTGS2* expression through molecular binding, reverse ferroptosis in BMSCs, and restore osteogenic ability (80). Baicalein (BN) significantly downregulates *PTGS2* protein in bone tissue in the OVX rat model, cooperatively regulates the SIRT1/AR/ESR1 pathway to improve bone loss (59). The active components of elderberry (such as kaempferol, quercetin) inhibit *PTGS2* and activate the PI3K–Akt pathway to promote bone matrix calcification (79). It is worth noting that the physiological necessity of *PTGS2* in the reproductive system suggests that its inhibitors should also have bone-targeting specificity (77). In summary, *PTGS2*, as a key target for OPC and a dual driver of “inflammation-bone resorption activation” and “ferroptosis-bone formation inhibition” in the process of OP, has become a common target for various natural anti-OP drugs.

On the other hand, *NR4A1* (Nuclear Receptor Subfamily 4 Group A Member 1, also known as Nur77) is an orphan nuclear receptor that plays a central regulatory role in postmenopausal OP through multiple mechanisms (81). It inhibits the differentiation of BMSCs into osteoblasts while promoting their differentiation into adipocytes, leading to increased bone marrow adiposity and reduced bone formation (60, 81, 82). Additionally, as a negative regulator of osteopontin, the loss of *NR4A1* enhances osteopontin-mediated migration and recruitment of osteoclast precursors to the bone surface, thereby accelerating trabecular bone resorption (81, 83). In contrast, pharmacological activation of *NR4A1* can suppress this process and slow bone loss (84). Under oxidative stress conditions, *NR4A1* influences osteoblast function by regulating autophagic flux; its downregulation exacerbates autophagy blockage, further impairing bone formation (60). Therefore, by disrupting the balance between bone formation and resorption, promoting bone marrow adipogenesis, and responding to oxidative stress, *NR4A1* has emerged as a critical therapeutic target in postmenopausal OP.

Our experimental results show that OPC effectively inhibits the expression of *PTGS2* by interfering with the transcriptional regulation mediated by *NR4A1*. In animal model studies, we not only observed the significant improvement effect of OPC on bone microstructure but also found that it could coordinate the regulation of bone turnover markers and restore bone metabolic balance. Mechanism studies indicate that this protective effect of OPC depends on the β-catenin-mediated *NR4A1*–*Runx2* signaling axis. This finding offers a fresh molecular viewpoint for comprehending OPC’s mode of action. Overall, OPC exerts therapeutic effects through a multilevel regulatory network, providing a new paradigm for natural products to target subpopulations of BMSCs to improve bone homeostasis.

However, this study still has several limitations that need attention: Firstly, although the OVX mouse model mimics some characteristics of postmenopausal OP to a certain extent, it still has significant differences from the complex pathophysiological process of human diseases. In particular, factors such as age, gender, and comorbidities were not considered, which may limit the clinical inference of the research results. Secondly, the research mainly focuses on MSCs and their subtypes, but the interaction mechanisms with other cell types in the bone microenvironment (like osteoclast precursors and immune cells) have not been adequately explored. Additionally, the current sample size is small, and *in vitro* experiments cannot fully replicate the complex physiological or microenvironmental conditions *in vivo*. At the clinical translation level, the optimal administration regimen of OPCs, their long-term safety, and potential synergistic effects with existing anti-OP drugs are still unclear, and natural products have inherent limitations in bioavailability and the long-term medication requirements for chronic diseases. Therefore, the current conclusions still need to be verified in large animal models with larger sample sizes and subsequent clinical trials. Future studies should concentrate on creating targeted treatment plans using MSC subtype molecular typing, optimizing the delivery system of OPC to enhance its bone targeting, and exploring its synergistic combination effects with other natural active components. These in-depth explorations will provide more universal scientific evidence supporting the clinical practice of OPC.

## Conclusion

In summary, our research indicates that OPC may serve as a potential therapeutic candidate by targeting and regulating C2 *NR4A1*+ MSCs in osteopenia, thereby playing a role in the prevention and treatment of OP. Specifically, it achieves a synergistic effect of “osteoclast inhibition and osteoblast promotion” through a dual signaling axis of *NR4A1*-*PTGS2* and β-catenin-dependent *NR4A1*–*Runx2*. This unique regulatory pattern not only explains the mechanism of OPC in preventing and treating OP but also provides new potential therapeutic targets for the development of OPC-based OP treatment strategies. Future research should focus on exploring the clinical translation pathways of OPC, including optimizing the administration methods, exploring its combined application with existing anti-OP drugs, and developing targeted delivery strategies for the bone microenvironment. These efforts will provide more practical guidance for the application of OPC in the prevention and treatment of OP and lay the foundation for subsequent clinical research.

## Data Availability

The original contributions presented in the study are included in the article/**Supplementary Material**. Further inquiries can be directed to the corresponding author.

## Supplementary material

Supplementary Figure 1 PPI network and enrichment analysis results. **(A)**PPI network. **(B, C)** GO (upper) and KEGG (lower) term networks in ClueGO.

Supplementary Table 1 Gene-specific primers.
