# Supplementary figures and images for: Deciphering oligomeric proanthocyanidins’ dual osteoprotective mechanisms at single-cell resolution: NR4A1-mediated PTGS2 suppression and β-catenin-Runx2 activation
Source: Front Immunol. 2025 Nov 4;16:1679987. doi: 10.3389/fimmu.2025.1679987 (PMC12623368; doi:10.3389/fimmu.2025.1679987)

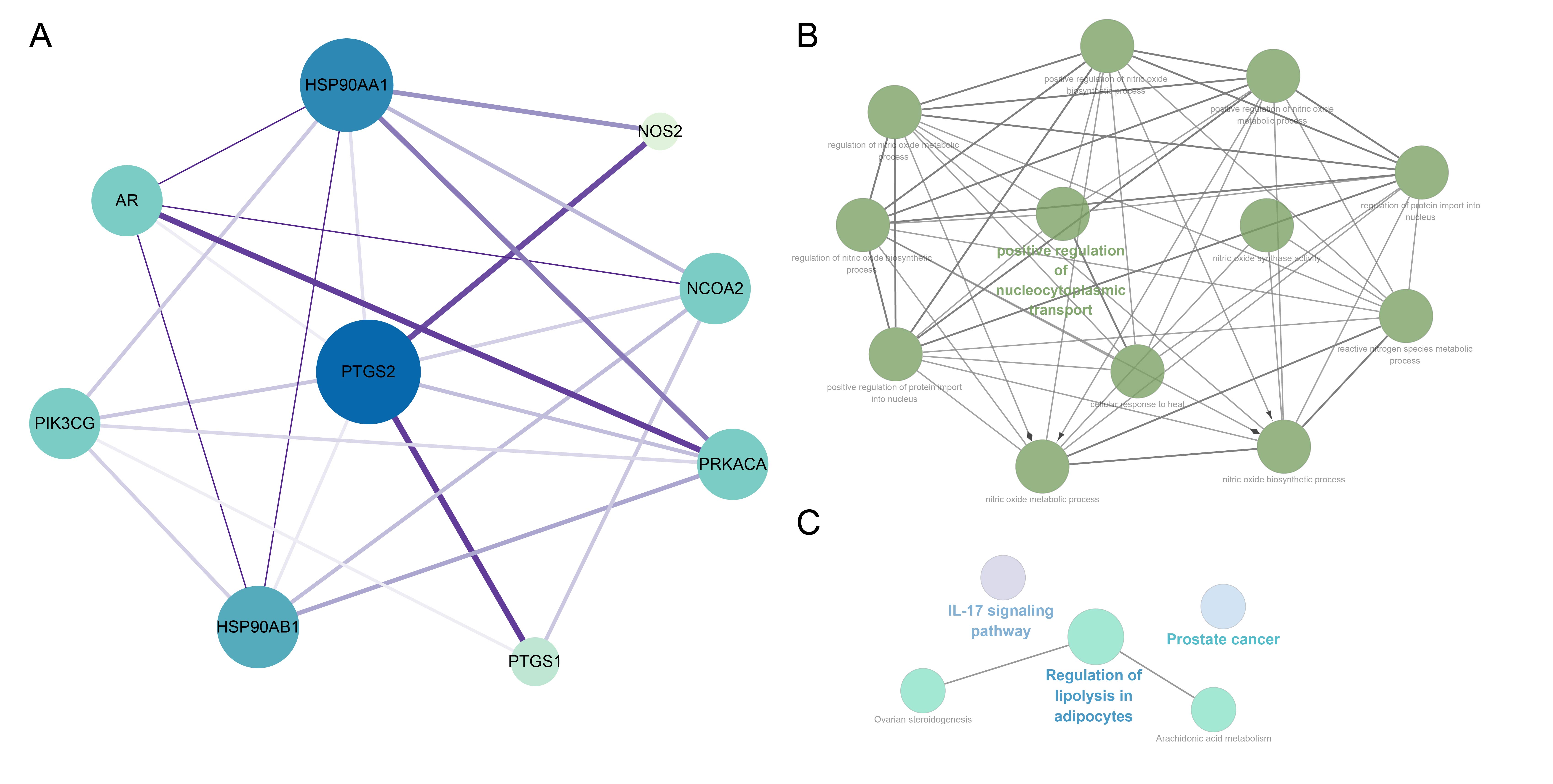

Supplement: Supplementary Figure 1 — PPI network and enrichment analysis results. (A)PPI network. (B, C) GO (upper) and KEGG (lower) term networks in ClueGO. [file Image1.jpeg]
