# Supplementary material for: Deciphering oligomeric proanthocyanidins’ dual osteoprotective mechanisms at single-cell resolution: NR4A1-mediated PTGS2 suppression and β-catenin-Runx2 activation
Source: Front Immunol. 2025 Nov 4;16:1679987. doi: 10.3389/fimmu.2025.1679987 (PMC12623368; doi:10.3389/fimmu.2025.1679987)
Supplement: Supplementary Table 1 — Gene-specific primers. [file Table1.docx]

| **Gene Symbol** | **Full Name** | **Direction** | **Primer Sequence (5'→3')** | **Amplicon (bp)** | **Exon Span** | **Accession #** |
| --- | --- | --- | --- | --- | --- | --- |
| **PTGS2** | Prostaglandin-endoperoxide synthase 2 (COX-2) | Forward | TTCAAATGAGATTGTGGGAAAATTG | 108 | 6-7 | NM_000963 |
|  |  | Reverse | AGATCATCTCTGCCTGAGTATCTT |  |  |  |
| **NR4A1** | Nuclear receptor subfamily 4 group A member 1 | Forward | CAGCAACCTCTGCCTCAAC | 115 | 3-4 | NM_002135 |
|  |  | Reverse | GCTGGTGTTGAGGTCGTAGTT |  |  |  |
| **TNFSF11** | TNF superfamily member 11 (RANKL) | Forward | CAGCATCAAAATACAGAGCGACAG | 95 | 2-3 | NM_003701 |
|  |  | Reverse | TCCATATGGACCAGCAAGGTT |  |  |  |
| **TNFRSF11B** | TNF receptor superfamily member 11B (OPG) | Forward | TGAAACCCCAGAGCGAAACA | 105 | 3-4 | NM_002546 |
|  |  | Reverse | GGCACACAGGGACATCTTTG |  |  |  |
| **RUNX2** | Runt-related transcription factor 2 | Forward | CCGCACGACAACCGCACCAT | 120 | 5-6 | NM_001015051 |
|  |  | Reverse | CGCTCCGGCCCACAAATCTC |  |  |  |
| **BGLAP** | Osteocalcin (Bone gamma-carboxyglutamate protein) | Forward | CATGAGAGCCCTCACACTCCTC | 98 | 2-3 | NM_199173 |
|  |  | Reverse | AGAGCGACACCCTAGACGGG |  |  |  |
